# Supplementary material for: Impact of Preexisting Rare Diseases on COVID-19 Severity, Reinfection, and Long COVID, and the Modifying Effects of Vaccination and Antiviral Therapy: A Retrospective Study from the N3C Data Enclave
Source: medRxiv. 2025 Jul 10:2025.07.09.25331138. Preprint. [Version 1] doi: 10.1101/2025.07.09.25331138 (PMC12265750; doi:10.1101/2025.07.09.25331138)
Supplement: Supplement 1 [file media-1.pdf]

Impact of Preexisting Rare Diseases on COVID-19 Severity, Reinfection, and Long COVID, and the Modifying Effects of Vaccination and Antiviral Therapy: A Retrospective Study from the N3C Data Enclave

Arjun S. Yadaw[1], David K Sahner[4], Eric Sid[3], Emily Y. Chew[2], Dominique Pichard[3], Ewy A. Mathé[1], On behalf the N3C Consortium

[1]Division of Preclinical Innovation, National Center for Advancing Translational Sciences (NCATS), NIH, Rockville, MD, USA
[2]Division of Epidemiology and Clinical Applications, National Eye Institute, National Institutes of Health, Bethesda, MD, USA
[3]Division of Rare Diseases Research Innovation, National Center for Advancing Translational Sciences (NCATS), National Institutes of Health, Bethesda, MD, USA.
[4]Former Senior Advisor, National Center for Advancing Translational Sciences (NCATS), NIH, Rockville, MD, USA

Univariate & multivariate models adjusted for demographics and comorbidities (Table 2/S1)

Univariate model: (Severity ~ Rare disease(i)),
Multivariate model: (Severity ~~ Rare disease(i)+ Demographic + Comorbidities); i = 1,…,18.

A Multivariable Model Evaluating the Impact of Vaccination and Antiviral Therapy on COVID-19 Severity by Rare Disease Status (*Table3/S3*)
{Treatment (vaccination and/or antiviral treatment) vs control}

Severity ~ treatment + demographic + comorbidities

A Multivariable Model Evaluating the Impact of Vaccination and Antiviral Therapy on Long COVID by Rare Disease Status (*Table4*)
{Treatment (vaccination and/or antiviral treatment) vs control}

Long COVID ~ treatment + demographic + comorbidities

A Multivariable Model Evaluating the Impact of Vaccination on COVID reinfection by Rare Disease Status (*Table5*)
{Treatment (vaccination vs control)}

COVID Reinfection ~ treatment + demographic + comorbidities

Figure S1: Statistical models evaluated among COVID-19 patients in the N3C to assess associations between preexisting rare diseases (RDs) and three outcomes: COVID-19 severity, long COVID, and reinfection. Severity outcomes include hospitalization (Yes/No) and life-threatening illness (Yes/No). Similar binary definitions are used for long COVID and reinfection outcomes. Models are adjusted for demographic covariates and preexisting comorbidities (see Methods).

Table S1: Univariate logistic regression models of severity outcomes.

	Life-threatening Yes: 113,510 No: 4,712,095			Hospitalized Yes: 254,728 No: 4,570,877	
	Univariate OR (95% CI)	*P* value		Univariate OR (95% CI)	*P* value
Rare neoplastic disease	5.12 (4.95 - 5.31)	<0.001	Rare endocrine disease	3.27 (3.0 - 3.56)	<0.001
Rare respiratory disease	4.69 (4.52 - 4.87)	<0.001	Rare respiratory disease	3.2 (3.1 - 3.3)	<0.001
Rare endocrine disease	3.42 (3.04 - 3.85)	<0.001	Rare hematologic disease	3.06 (2.97 - 3.15)	<0.001
Rare gastroenterologic disease	3.37 (3.11 - 3.66)	<0.001	Rare neoplastic disease	2.66 (2.58 - 2.75)	<0.001
Rare hepatic disease	3.18 (2.92 - 3.46)	<0.001	Rare gastroenterologic disease	2.56 (2.4 - 2.73)	<0.001
Rare hematologic disease	2.94 (2.82 - 3.07)	<0.001	Rare bone diseases	2.36 (2.15 - 2.6)	<0.001
Rare bone diseases	2.92 (2.58 - 3.31)	<0.001	Rare renal disease	2.31 (2.07 - 2.58)	<0.001
Rare infectious disease	2.78 (2.62 - 2.95)	<0.001	Rare ophthalmic disorder	2.3 (2.16 - 2.45)	<0.001
Rare ophthalmic disorder	2.57 (2.36 - 2.8)	<0.001	Rare hepatic disease	2.28 (2.13 - 2.44)	<0.001
Rare systemic or rheumatologic disease	2.56 (2.46 - 2.66)	<0.001	Rare systemic or rheumatologic disease	2.19 (2.13 - 2.26)	<0.001
Rare cardiac diseases	2.51 (2.17 - 2.9)	<0.001	Rare infectious disease	2.16 (2.07 - 2.27)	<0.001
Rare neurologic disease	2.39 (2.31 - 2.46)	<0.001	Rare cardiac diseases	2.14 (1.92 - 2.39)	<0.001
Rare immune disease	2.24 (2.04 - 2.45)	<0.001	Rare neurologic disease	2.11 (2.06 - 2.16)	<0.001
Rare renal disease	1.97 (1.67 - 2.33)	<0.001	Rare developmental defect during embryogenesis	2.04 (1.96 - 2.13)	<0.001
Rare skin disease	1.93 (1.78 - 2.1)	<0.001	Rare immune disease	1.81 (1.69 - 1.94)	<0.001
Rare developmental defect during embryogenesis	1.75 (1.65 - 1.87)	<0.001	Rare otorhinolaryngologic disease	1.72 (1.56 - 1.9)	<0.001
Rare otorhinolaryngologic disease	1.67 (1.44 - 1.93)	<0.001	Rare inborn errors of metabolism	1.57 (1.43 - 1.72)	<0.001
Rare inborn errors of metabolism	1.45 (1.26 - 1.67)	<0.001	Rare skin disease	1.52 (1.43 - 1.62)	<0.001

Table S2: Characteristics of COVID-19 subcohort of patients with reliable treatment documentation (December 23rd, 2021, to Jan 4th, 2024, n = 799,662) for assessing the impact of vaccination and antiviral treatment.

	All patients (N = 799,662)	Rare Disease (No) (N = 733,641)	Rare disease (Yes) (N = 66,021)
**COVID-19 prevention/intervention**			
Antiviral treatment, n (%)	42,023 (5.3%)	38,385 (5.2%)	3,638 (5.5%)
Vaccination status, n (%)	321,834 (40%)	294,607 (40%)	27,227 (41%)
Antiviral & vaccination, n (%)	129,727 (16%)	117,079 (16%)	12,648 (19%)
Control, n (%)	306,078 (38%)	283,570 (39%)	22,508 (34%)
**COVID-19 related outcomes**			
Life-threatening, n (%)	16,712 (2.1%)	13,443 (1.8%)	3,269 (5.0%)
Hospitalized, n (%)	39,464 (4.9%)	33,161 (4.5%)	6,303 (9.5%)
Long COVID, n (%)	46,494 (5.8%)	39,263 (5.4%)	7,231 (11%)
**Demographic**			
Age, n (%)			
Age (1-20)	93,389 (12%)	88,385 (12%)	5,004 (7.6%)
Age (20-40)	192,475 (24%)	180,839 (25%)	11,636 (18%)
Age (40-65)	298,635 (37%)	274,026 (37%)	24,609 (37%)
Age (>65)	215,163 (27%)	190,391 (26%)	24,772 (38%)
BMI, n (%)			
Obese	437,153 (55%)	400,396 (55%)	36,757 (56%)
Over weight	197,732 (25%)	181,155 (25%)	16,577 (25%)
Normal	130,938 (16%)	120,544 (16%)	10,394 (16%)
Under weight	33,839 (4.2%)	31,546 (4.3%)	2,293 (3.5%)
Sex, n(%)			
Female	493,441 (62%)	451,950 (62%)	41,491 (63%)
Male	306,221 (38%)	281,691 (38%)	24,530 (37%)
Race, n(%)			
Asian	41,367 (5.2%)	38,846 (5.3%)	2,521 (3.8%)
Black or African American	105,988 (13%)	96,045 (13%)	9,943 (15%)
Missing/Unknown/Other	93,058 (12%)	87,287 (12%)	5,771 (8.7%)
White	559,249 (70%)	511,463 (70%)	47,786 (72%)
Ethinicity, n(%)			
Hispanic or Latino	99,047 (12%)	94,211 (13%)	4,836 (7.3%)
Not hispanic or Latino	670,302 (84%)	610,705 (83%)	59,597 (90%)
Missing/Unknown	30,313 (3.8%)	28,725 (3.9%)	1,588 (2.4%)
Smoking status, n(%)			
Current or former smoker	138,643 (17%)	125,550 (17%)	13,093 (20%)
Non-smoker	661,019 (83%)	608,091 (83%)	52,928 (80%)
**Comorbidities, n(%)**			
Cancer	92,773 (12%)	74,629 (10%)	18,144 (27%)
Cardiomyophaties	27,141 (3.4%)	21,661 (3.0%)	5,480 (8.3%)
Cerebro vascular disease	45,575 (5.7%)	36,958 (5.0%)	8,617 (13%)
Chronic lung disease	179,477 (22%)	152,497 (21%)	26,980 (41%)
Coronary artery disease	79,411 (9.9%)	66,780 (9.1%)	12,631 (19%)
Dementia before	20,088 (2.5%)	17,073 (2.3%)	3,015 (4.6%)
Depression	182,887 (23%)	160,081 (22%)	22,806 (35%)
Diabetes	142,647 (18%)	125,383 (17%)	17,264 (26%)
Heart failure	60,220 (7.5%)	48,172 (6.6%)	12,048 (18%)
HIV infection	6,144 (0.8%)	5,208 (0.7%)	936 (1.4%)
Hypertension	310,976 (39%)	274,323 (37%)	36,653 (56%)
Kidney disease	81,261 (10%)	66,679 (9.1%)	14,582 (22%)
Liver disease	61,810 (7.7%)	50,261 (6.9%)	11,549 (17%)
Myocardial infraction	34,945 (4.4%)	28,716 (3.9%)	6,229 (9.4%)
Peripheral vascular disease	38,007 (4.8%)	31,268 (4.3%)	6,739 (10%)
Rheumatologic disease	64,127 (8.0%)	49,659 (6.8%)	14,468 (22%)

Table S3: Impact of vaccination and antiviral treatment exposures on hospitalization in patients with and without preexising RDs (multivariate logistic regression models are adjusted for demographics and comorbidities).

	Patients with Preexisting Rare Disease						Patients without Preexisting Rare Disease					
	Vaccination vs Control		Antiviral treatment vs Control		Vaccination + antiviral treatment vs Control		Vaccination vs Control		Antiviral treatment vs Control		Vaccination + antiviral treatment vs Control	
	Hospitalized condition Yes: 5,888 No: 43,847		Hospitalized condition Yes: 3,192 No: 22,954		Hospitalized condition Yes: 6,303 No: 59,718		Hospitalized condition Yes: 30,958 No: 547,219		Hospitalized condition Yes: 19,727 No: 302,228		Hospitalized condition Yes: 33,161 No: 700,480	
	**OR (95% CI)**	**P value**	**OR (95% CI)**	**P value**	**OR (95% CI)**	**P value**	**OR (95% CI)**	**P value**	**OR (95% CI)**	**P value**	**OR (95% CI)**	**P value**
**-19 prevention/intervention**												
rol	1 [Reference]		1 [Reference]		1 [Reference]		1 [Reference]		1 [Reference]		1 [Reference]	
Vaccination status	**0.62 (0.59 - 0.66)**	**< 0.001**					**0.45 (0.44 - 0.47)**	**< 0.001**				
Antiviral treatment			**0.31 (0.26 - 0.37)**	**< 0.001**					**0.34 (0.31 - 0.36)**	**< 0.001**		
Vaccination & Antiviral					**0.11 (0.1 - 0.13)**	**< 0.001**					**0.09 (0.09 - 0.1)**	**< 0.001**
**Demographic**												
**Age**												
Age (20-40)	1 [Reference]		1 [Reference]		1 [Reference]		1 [Reference]		1 [Reference]		1 [Reference]	
Age (1-20)	1.05 (0.9 - 1.24)	0.540	0.89 (0.75 - 1.05)	0.167	0.92 (0.77 - 1.09)	0.312	0.48 (0.45 - 0.52)	< 0.001	0.5 (0.47 - 0.54)	< 0.001	0.48 (0.44 - 0.52)	< 0.001
Age (40-65)	0.93 (0.84 - 1.02)	0.121	1.04 (0.92 - 1.17)	0.498	1.01 (0.9 - 1.14)	0.811	1.02 (0.99 - 1.07)	0.225	1.02 (0.97 - 1.06)	0.470	1.01 (0.96 - 1.05)	0.808
Age (>65)	1.22 (1.1 - 1.36)	< 0.001	1.27 (1.11 - 1.46)	0.001	1.27 (1.11 - 1.45)	0.001	1.85 (1.77 - 1.94)	< 0.001	1.83 (1.73 - 1.93)	< 0.001	1.78 (1.69 - 1.89)	< 0.001
**BMI**												
Normal	1 [Reference]		1 [Reference]		1 [Reference]		1 [Reference]		1 [Reference]		1 [Reference]	
Obese	0.75 (0.69 - 0.82)	< 0.001	0.72 (0.64 - 0.81)	< 0.001	0.71 (0.63 - 0.8)	< 0.001	0.72 (0.7 - 0.75)	< 0.001	0.76 (0.72 - 0.79)	< 0.001	0.71 (0.68 - 0.75)	< 0.001
Over weight	0.77 (0.7 - 0.85)	< 0.001	0.8 (0.7 - 0.91)	0.001	0.77 (0.68 - 0.88)	< 0.001	0.83 (0.8 - 0.87)	< 0.001	0.87 (0.82 - 0.91)	< 0.001	0.81 (0.77 - 0.86)	< 0.001
Under weight	0.79 (0.63 - 0.98)	0.030	0.76 (0.61 - 0.94)	0.011	0.8 (0.64 - 1)	0.050	0.82 (0.74 - 0.91)	< 0.001	0.7 (0.63 - 0.78)	< 0.001	0.71 (0.64 - 0.79)	< 0.001
**Sex**												
Female	1 [Reference]		1 [Reference]		1 [Reference]		1 [Reference]		1 [Reference]		1 [Reference]	
Male	1.3 (1.22 - 1.38)	< 0.001	1.32 (1.21 - 1.43)	< 0.001	1.29 (1.19 - 1.41)	< 0.001	1.17 (1.14 - 1.2)	< 0.001	1.13 (1.09 - 1.16)	< 0.001	1.16 (1.12 - 1.2)	< 0.001
**Race**												
White	1 [Reference]		1 [Reference]		1 [Reference]		1 [Reference]		1 [Reference]		1 [Reference]	
Asian	1.01 (0.86 - 1.2)	0.887	1.01 (0.81 - 1.26)	0.937	0.99 (0.78 - 1.24)	0.905	0.9 (0.85 - 0.96)	0.002	0.91 (0.84 - 0.98)	0.016	0.93 (0.85 - 1.01)	0.077
Black or African American	1.67 (1.54 - 1.8)	< 0.001	1.58 (1.42 - 1.76)	< 0.001	1.56 (1.4 - 1.74)	< 0.001	1.36 (1.31 - 1.41)	< 0.001	1.4 (1.34 - 1.47)	< 0.001	1.39 (1.33 - 1.45)	< 0.001
Missing/Unknown/Other	1.2 (1.06 - 1.35)	0.004	1.17 (1 - 1.35)	0.043	1.21 (1.04 - 1.41)	0.014	1.17 (1.11 - 1.22)	< 0.001	1.13 (1.07 - 1.2)	< 0.001	1.15 (1.09 - 1.22)	< 0.001
**Ethinicity**												
Not hispanic or Latino	1 [Reference]		1 [Reference]		1 [Reference]		1 [Reference]		1 [Reference]		1 [Reference]	
Hispanic or Latino	1.19 (1.04 - 1.36)	0.009	1.25 (1.07 - 1.47)	0.005	1.2 (1.02 - 1.42)	0.029	0.8 (0.76 - 0.84)	< 0.001	0.9 (0.85 - 0.95)	< 0.001	0.9 (0.84 - 0.96)	0.001
Missing/Unknown	0.45 (0.34 - 0.6)	< 0.001	0.45 (0.31 - 0.64)	< 0.001	0.42 (0.29 - 0.61)	< 0.001	0.46 (0.42 - 0.51)	< 0.001	0.51 (0.45 - 0.57)	< 0.001	0.47 (0.41 - 0.53)	< 0.001
**Smoking status**												
Non-smoker	1 [Reference]		1 [Reference]		1 [Reference]		1 [Reference]		1 [Reference]		1 [Reference]	
Current or former smoker	1.42 (1.32 - 1.53)	< 0.001	1.15 (1.04 - 1.28)	0.005	1.18 (1.07 - 1.31)	0.001	1.39 (1.35 - 1.44)	< 0.001	1.18 (1.14 - 1.23)	< 0.001	1.19 (1.14 - 1.24)	< 0.001
**Comorbidities**												
cer	1.34 (1.25 - 1.43)	< 0.001	1.31 (1.2 - 1.45)	< 0.001	1.35 (1.22 - 1.48)	< 0.001	1.31 (1.26 - 1.35)	< 0.001	1.29 (1.23 - 1.35)	< 0.001	1.29 (1.23 - 1.36)	< 0.001
liomyophaties	0.97 (0.87 - 1.07)	0.534	0.87 (0.75 - 1.01)	0.069	0.89 (0.77 - 1.03)	0.126	1.02 (0.96 - 1.08)	0.520	1 (0.93 - 1.08)	0.897	1.01 (0.93 - 1.08)	0.873
ebro vascular disease	1.26 (1.17 - 1.37)	< 0.001	1.28 (1.14 - 1.44)	< 0.001	1.3 (1.16 - 1.46)	< 0.001	1.32 (1.26 - 1.37)	< 0.001	1.32 (1.25 - 1.39)	< 0.001	1.34 (1.26 - 1.41)	< 0.001
nic lung disease	1.37 (1.29 - 1.46)	< 0.001	1.43 (1.31 - 1.55)	< 0.001	1.4 (1.28 - 1.52)	< 0.001	1.46 (1.42 - 1.51)	< 0.001	1.44 (1.39 - 1.5)	< 0.001	1.49 (1.44 - 1.55)	< 0.001
nary artery disease	1.03 (0.94 - 1.12)	0.567	0.97 (0.86 - 1.1)	0.630	0.98 (0.87 - 1.1)	0.711	1.03 (0.99 - 1.08)	0.134	1 (0.95 - 1.06)	0.923	1.01 (0.96 - 1.07)	0.639
entia before	1.63 (1.45 - 1.82)	< 0.001	1.37 (1.16 - 1.61)	< 0.001	1.48 (1.25 - 1.74)	< 0.001	2.22 (2.11 - 2.33)	< 0.001	2.02 (1.89 - 2.16)	< 0.001	2.13 (1.99 - 2.28)	< 0.001
ression	1.08 (1.01 - 1.15)	0.026	1.11 (1.01 - 1.22)	0.024	1.11 (1.01 - 1.21)	0.029	1.11 (1.08 - 1.14)	< 0.001	1.13 (1.09 - 1.18)	0.000	1.13 (1.08 - 1.18)	< 0.001
etes	1.25 (1.17 - 1.34)	< 0.001	1.29 (1.17 - 1.42)	< 0.001	1.27 (1.15 - 1.4)	< 0.001	1.39 (1.35 - 1.44)	< 0.001	1.4 (1.35 - 1.46)	< 0.001	1.41 (1.35 - 1.47)	< 0.001
t failure	1.61 (1.48 - 1.76)	< 0.001	1.5 (1.33 - 1.7)	< 0.001	1.54 (1.37 - 1.74)	< 0.001	1.93 (1.85 - 2.02)	< 0.001	1.8 (1.7 - 1.91)	< 0.001	1.8 (1.7 - 1.91)	< 0.001
infection	1.01 (0.8 - 1.28)	0.905	1.06 (0.8 - 1.42)	0.678	1.09 (0.8 - 1.47)	0.595	1.19 (1.03 - 1.37)	0.015	1.32 (1.11 - 1.57)	0.002	1.26 (1.05 - 1.51)	0.013
Hypertension	1.41 (1.3 - 1.54)	< 0.001	1.47 (1.32 - 1.64)	< 0.001	1.49 (1.34 - 1.67)	< 0.001	1.62 (1.56 - 1.67)	< 0.001	1.59 (1.52 - 1.66)	< 0.001	1.58 (1.51 - 1.65)	< 0.001
ey disease	1.64 (1.53 - 1.76)	< 0.001	1.61 (1.46 - 1.77)	< 0.001	1.62 (1.47 - 1.78)	< 2e-16	1.71 (1.65 - 1.77)	< 0.001	1.66 (1.59 - 1.74)	< 0.001	1.64 (1.57 - 1.72)	< 0.001
disease	1.22 (1.14 - 1.32)	< 0.001	1.28 (1.16 - 1.42)	< 0.001	1.3 (1.17 - 1.44)	< 0.001	1.39 (1.34 - 1.45)	< 0.001	1.44 (1.36 - 1.51)	< 0.001	1.4 (1.32 - 1.48)	< 0.001
cardial infraction	1.25 (1.14 - 1.37)	< 0.001	1.3 (1.15 - 1.49)	< 0.001	1.33 (1.17 - 1.52)	< 0.001	1.55 (1.47 - 1.62)	< 0.001	1.56 (1.47 - 1.66)	< 0.001	1.54 (1.45 - 1.63)	< 0.001
heral vascular disease	1.11 (1.01 - 1.21)	0.025	1.04 (0.92 - 1.18)	0.537	1.06 (0.94 - 1.21)	0.346	1.06 (1.02 - 1.11)	0.008	1.03 (0.96 - 1.09)	0.429	1.05 (0.98 - 1.11)	0.147
matologic disease	0.96 (0.9 - 1.03)	0.277	0.85 (0.77 - 0.94)	0.002	0.87 (0.78 - 0.96)	0.006	1.03 (0.99 - 1.08)	0.117	0.99 (0.93 - 1.04)	0.611	1.02 (0.97 - 1.08)	0.464
